# Developing a Penetrometer-Based Mapping System for Visualizing Silage Bulk Density from the Bunker Silo Face

**DOI:** 10.3390/s16071038

**Published:** 2016-07-05

**Authors:** Menghua Li, Kerstin H. Jungbluth, Yurui Sun, Qiang Cheng, Christian Maack, Wolfgang Buescher, Jianhui Lin, Haiyang Zhou, Zhongyi Wang

**Affiliations:** 1 College of Information and Electrical Engineering, China Agricultural University, Key Lab of Agricultural Information Acquisition Technology, Ministry of Agriculture, 100083 Beijing, China; lmh@cau.edu.com (M.L.); zhouhy@cau.edu.cn (H.Z.); wzyhl@cau.edu.cn (Z.W.); 2 Department of Agricultural Engineering, The University of Bonn, 53115 Bonn, Germany; kjungblu@uni-bonn.de (K.H.J.); c.maack@uni-bonn.de (C.M.); buescher@uni-bonn.de (W.B.); 3 School of Technology, Beijing Forestry University, 100083 Beijing, China; swiq_lin@163.com

**Keywords:** bunker silo, silage, bulk density, penetrometer, measurement, mapping

## Abstract

For silage production, high bulk density (BD) is critical to minimize aerobic deterioration facilitated by oxygen intrusion. To precisely assess packing quality for bunker silos, there is a desire to visualize the BD distribution within the silage. In this study, a penetrometer-based mapping system was developed. The data processing included filtering of the penetration friction component (PFC) out of the penetration resistance (PR), transfer of the corrected penetration resistance (PR_c_) to BD, incorporation of Kriged interpolation for data expansion and map generation. The experiment was conducted in a maize bunker silo (width: 8 m, middle height: 3 m). The BD distributions near the bunker silo face were represented using two map groups, one related to horizontal- and the other to vertical-density distribution patterns. We also presented a comparison between the map-based BD results and core sampling data. Agreement between the two measurement approaches (RMSE = 19.175 kg·m^−3^) demonstrates that the developed penetrometer mapping system may be beneficial for rapid assessment of aerobic deterioration potential in bunker silos.

## 1. Introduction

Bunker silos are recommended for dairy-farm scales of 100 cows or more when the silo is unloaded at feeding rates above 100 mm·d^−1^ in summer and 75 mm·d^−1^ in winter. The merits of siloed feed include a relatively low storage cost, minimal loss of biomass and time-saving management [1,2]. On the other hand, there is a high risk of silage spoilage near the zone of the exposure face when a bunker silo is opened for livestock feeding. In this situation, the silo face is exposed to air; facilitating rapid growth of microorganisms and leading to aerobic deterioration as oxygen rapidly diffuses into the silage. Thus, it is critical for bunker silo management to maintain an optimal face-removal-rate associated with aerobic stability in the silage [3].

High silage bulk density (BD) can significantly reduce aerobic deterioration because the high BD creates low porosity, thereby reducing O_2_ diffusion into the silage [4,5,6,7]. Well-compacted silage should not only exhibit a high BD, but a uniform BD distribution as well [7]. In reality, the BD of maize silage can be highly variable at the farm scale in bunker silos. For instance, a previous study reported BD values that ranged from 125 to 378 kg·m^−^^3^ dry matter (DM) content for maize silage based on the investigation from 81 commercial bunker silos [8].

To assess the silage packing quality, a simple method was used to calculate the mean BD from the known packed mass and its volume. However, this approach does not reveal the spatial BD distribution within the silage. For map-based BD measurements, a gamma ray scanner was tested in two studies [9,10], where the relative measurement error was about ±1% after calibration. Despite the high accuracy, few producers would be able to effectively use gamma ray due to regulations and the potential danger of exposure to radiation. An improved penetrometer technique for map-based determination of BD in grass bale silage was developed [7]. Subsequently, a study verified that this novel technique can replace the gamma ray scanner for imaging silage BD distribution [11]. Considering that the spoilage risk for a bunker silo packed with maize silage is rather high [5], developing a penetrometer-based mapping system especially for maize silage in a bunker silo was the major objective of this study.

## 2. Materials and Methods

### 2.1. Penetrometer-Based Measurement Platform

**Figure 1:**
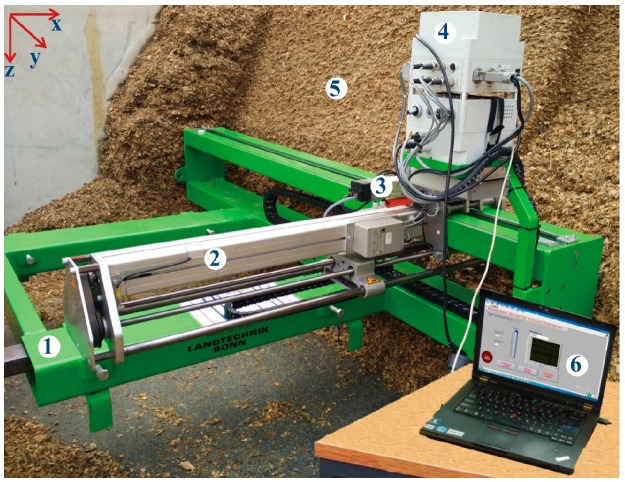
A photo of the penetrometer-based mapping system: (1) frame, (2) penetrometer, (3) motor for y-axis translation, (4) relay-box, (5) maize silo, and (6) the interface of the measurement system.

**Figure 2:**
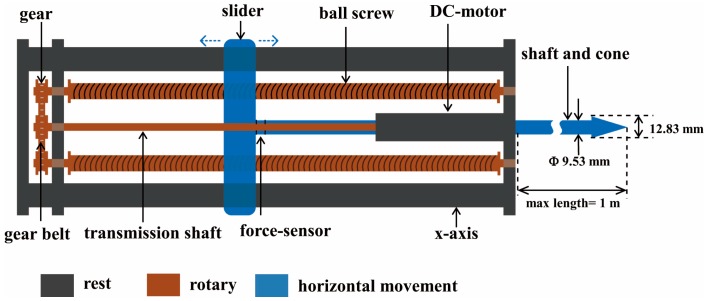
The mechanical structure and working principle of the penetrometer designed, where the black color refers to the rest part (frame), the brown color to the rotary part (crew-drive shafts), and blue color to the horizontal movement part (slide, penetration shaft and cone).

**Figure 3:**
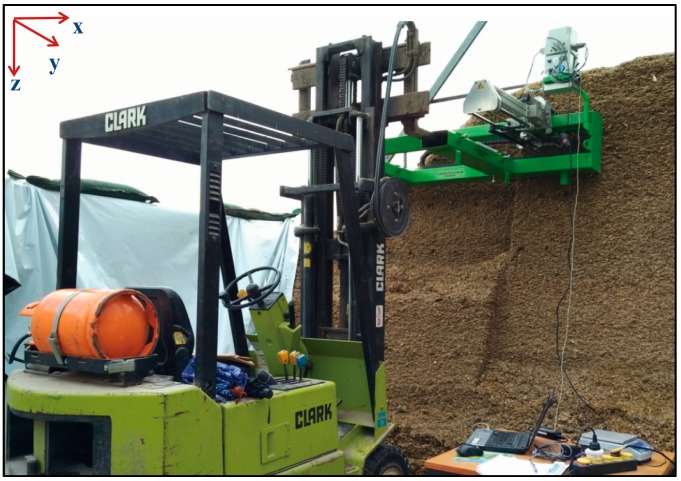
Using a forklift to position the frame prior to penetrating the face of the bunker silo at different heights.

Figure 1 shows the measurement platform made by us, consisting of a motorized penetrometer, a y-axis shifter driven by a brush motor (24 V, 200 W, 5930 rev. min^−1^, Maxon RE50, Sachseln, Switzerland) through a planetary gear device (reduction ratio, 57:11, Maxon GP62, Sachseln, Switzerland), a relay-box, all installed on a green steel-frame that mounts to a forklift device and facilitates vertical movement of the penetrometer mechanism parallel with the silage face. A LabVIEW-based measurement interface was programmed to control the measurement process using a laptop. Figure 2 illustrates the mechanical principle of the penetrometer, where the black color represents the penetrometer structural support (i.e., rest components), the brown color shows dual screw-drive shafts (i.e., rotary components) and the blue color illustrates the slide, penetration shaft and cone with linear movement function. The penetrometer was powered by a permanent-magnet synchronous motor (model M63x60/I, Kählig Antriebstechnik GmbH, Hannover, Germany, 12 V, 99 W maximum output power). Following the cone movement along *x*-axis, a potentiometer (ten-turn, 10 kΩ, ± 0.25% linearity) acted as a transducer to output the depth-specific signal. During the penetration process, when the cone reached the predetermined penetration depth (maximum measurement depth 1 m) or when the penetration resistance (PR) value exceeded 1000 N, the DC motor automatically reversed, causing the cone to retract to the original zero position. Based on Newton’s law of action and reaction, a constant cone velocity is reuqired because either acceleration or deceleration can cause uncertainty in the PR measurement [12,13,14]. To comply with American Society of Agricultural and Biological Engineers (ASABE) Standard S313.3 [15], the penetration velocity was controlled at 30 mm·s^−1^. Similarly, the dimension of penetration cone (diam. of the cone’s base 12.83 mm; cone apex 30°) and the shaft (diam. 9.53 mm) are designed based on the ASABE Standards [15,16,17]. In addition, Figure 3 shows that the entire apparatus deployed at the silage face with a forklift, which controlled the vertical (*z*-axis) positioning over a height of 3 m in 0.5 m increments.

### 2.2. Control Unit and LabVIEW-Based Interface

The control unit had three functions: (1) accomplishing a control sequence, (2) logging measurement data and (3) displaying results. To simplify the hardware design, an electronic multifunction module (USB-6212, National Instruments, Austin, TX, USA) was chosen which had 16 analog inputs (16-bit, 400 kHz), 2 analog outputs (16-bit, 250 kHz), 32 digital input/output channels (I/Os), and two 32-bit counters. A group of control cables connected the I/Os to a relay-box (Figure 1). The module used was compatible with LabVIEW (National Instruments, Austin, TX, USA), ANSI C/C++, C#, Visual Basic.Net and Visual Basic 6.0 software (Microsoft Corporation, St Redmond, WA, USA). The software was programmed with LabVIEW 6.0 (National Instruments, Austin, TX, USA) as a whole measurement process package following a logical sequence, except for the forklift positioning of the frame. Data acquired from each sensor were saved to a laptop as an EXCEL file and displayed graphically on interface. For instance, the PR results could be dynamically displayed as a curve or a hue bar associated with instant penetration depth on the relevant display panels as shown in Figure 1.

### 2.3. Data Processing Procedure

**Figure 4:**
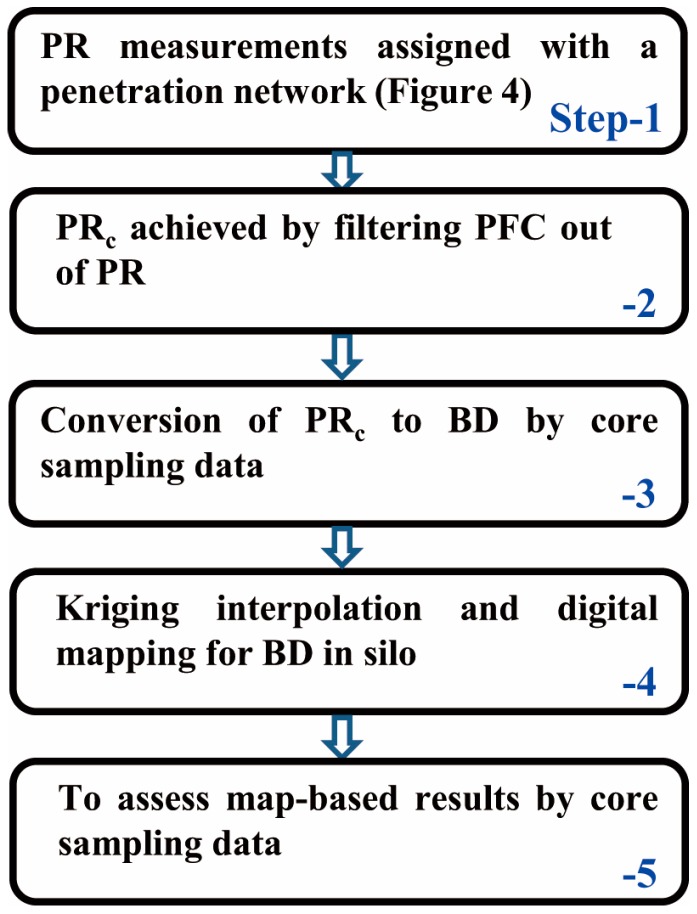
Flow chart of the penetration resistance data collection and processing procedures.

**Figure 5:**
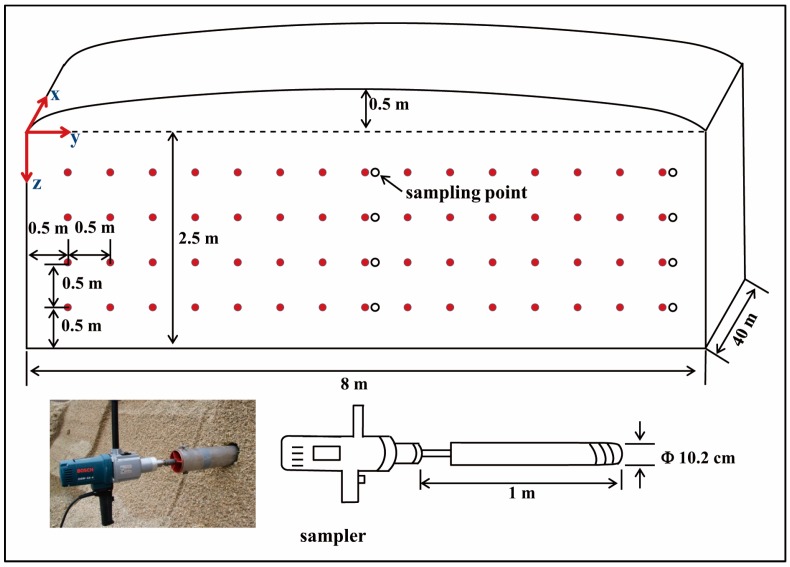
Measurement network showing core sampling location on the bunker silo face (solid dots) and the core sampler dimensions and locations (open circles).

Five steps listed in Figure 4 illustrate the PR data collection and processing for map generation of the silo silage density. Step-1 includes acquisition of PR measurements (*n* = 60) assigned with the penetration network (Figure 5) relative to a silo face (length 8 m, height 3 m).

**Figure 6:**
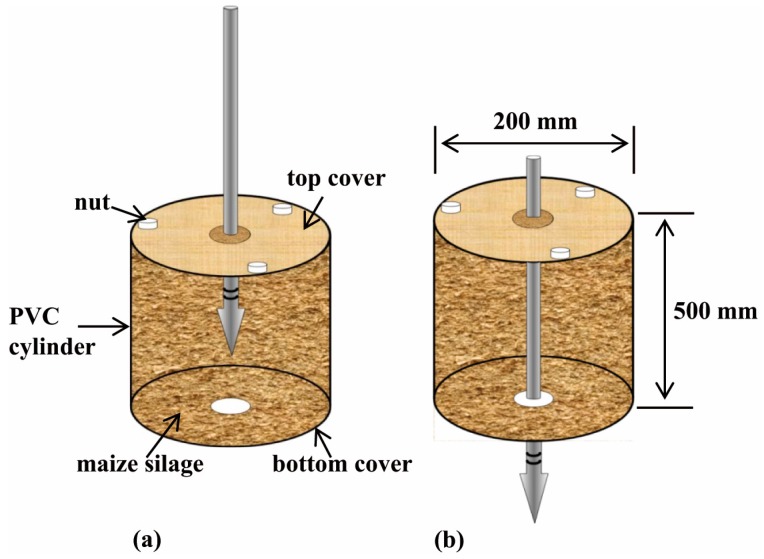
Determination of the penetration friction component (PFC) using a designed cylinder with two covers, each having a hole at the center. (**a**) the measurement for penetration resistance (PR) and, (**b**) the measurement for determining the penetration friction component (PFC).

Step-2 is to filter penetration friction out of the depth-related profile data. Previous studies have verified a substantial penetration friction force between the penetrometer shaft and maize silage being penetrated [18,19], creating uncertainty in how much of the PR should be translated as BD. The penetration friction component (PFC) was determined by penetrating a specific cylinder filled with maize silage at a known BD as illustrated in Figure 6. The cylinder (inside dia. 200 mm, height 500 mm) had two covers (dia. 200 mm, thickness 20 mm) and each cover had a hole (dia. 20 mm) at the center. Therefore, the penetration process included two phases. In phase-1 (Figure 6a), the PR measured was the sum of cone resistance (CR) and PFC. After the cone passed through the bottom of the cylinder (i.e., in phase-2; Figure 6b), the PR measured was only due to the PFC. As the literature stated [18], the PFC could be attributed to two factors: (1) it is directly proportional to the contact area of the shaft on the penetrating material, and (2) the overburden forces, and therefore the forces perpendicular to the shaft, increase as the penetration depth increases. Based on these, an approximate filter function (*f_c_*) was suggested as:

(1) $$f_{C}=\frac{C_{1}}{C_{2}+S_{shaft}}=\frac{C_{1}}{C_{2}+\pi D_{shaft}L_{depth}}$$

where *D_shaft_* denotes the contact area between the shaft and the maize silage, *C*_1_ and *C*_2_ are correction coefficients and are dependent on the elastic-plastic property of the measured material (*C*_1_ is a gain coefficient, and the initial filtering depends on the *C*_2_/*C*_1_ ratio), *L_depth_* is a dynamic parameter of penetration depth, and *D_shaft_* is the diameter of the shaft (9.53 mm). Thus, the corrected measurement value (*PR_c_*) can be calculated as the product of the instantly measured PR and *f_c_*:

(2) $$PR_{C}(L_{depth})=PR(L_{depth})f_{c}(L_{depth})=\frac{PR(L_{depth})C_{1}}{C_{2}+\pi D_{shaft}L_{depth}}\;100\;mm\leq L_{depth}$$

Moreover, for *m*-number of penetration profiles, *C*_1_ and *C*_2_ can be found using a pair of optimal solutions:

(3) $$\sigma {\left(C_{1},C_{2}\right)}^{2}=min\frac{1}{n}[\sum_{i=1}^{n}{\left(PR_{C}-PR\right)}_{i}^{2}]$$

and

(4) $$\{\begin{array}{c} \frac{\partial \sigma {\left(C_{1},C_{2}\right)}^{2}}{\partial C_{1}}=0\\ \frac{\partial \sigma {\left(C_{1},C_{2}\right)}^{2}}{\partial C_{2}}=0 \end{array}$$

After the PFC was filtered out of the PR measurements, the next task (i.e., Step-3) was to convert the PR_c_ to BD values using a transfer equation. For this, a core sampler (shown on the bottom of Figure 5) was used to extract maize silage samples. For each sampling process, two samples were extracted in 0.5 m increments of penetration depth. Here sampling data were randomly divided into two groups, half for determining the BD transfer equation and the other half for assessing map quality. The open circles in Figure 5 show the in situ BD sampling locations. All samples were weighed to determine the fresh/wet BD and then oven-dried for 24 h at 103 °C to determine silage moisture content [17]. In Step-4, two of the basic functions in ArcGIS 9.2 software were employed, the data post-conditioning by ordinary Kriging interpolation and the digital mapping with the expanded data set. As an unbiased estimation method to generate high-resolution maps, Kriging interpolation can optimally predict unknown values from the data measured at known locations associated with the spatial correlation of these data and the predicted variance. Finally, the map-based results were assessed using half of the core sampling data (Step-5).

### 2.4. Experimental Condition

**Figure 7:**
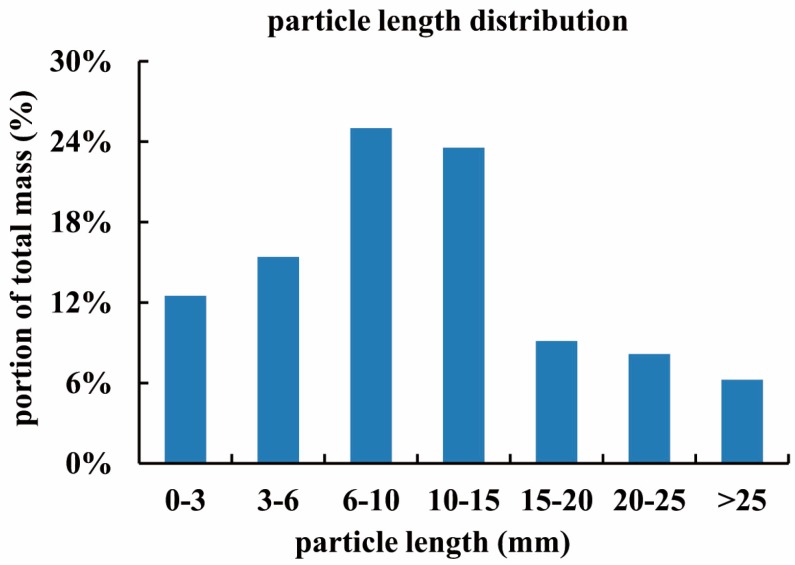
Chopped maize particle length distribution from the tested bunker silo.

The bunker silo (40 m × 8 m × 3 m), located at a dairy farm in Haus Riswick in Kleve, Germany, was constructed of two concrete side-walls and a back-wall. The maize crop filling the silo was harvested in the fall of 2014. Figure 7 illustrates the distribution of the chopped maize particle length. For compacting the bunker silo, a 12 ton tractor was used (Fendt Vario 714). A layer depth was 20 cm and the total packing time of the bunker silo was 12 h. The sampling data (*n* = 16) showed a mean DM of 335 kg·m^−3^. The measurement was conducted on 25 September 2015 when the silo was being unloaded at a rate of approximately 0.5 m per day. For the 60 penetration measurements shown in Figure 5, it took about 3 h.

## 3. Results and Discussion

### 3.1. Filtering PFC from PR

**Figure 8:**
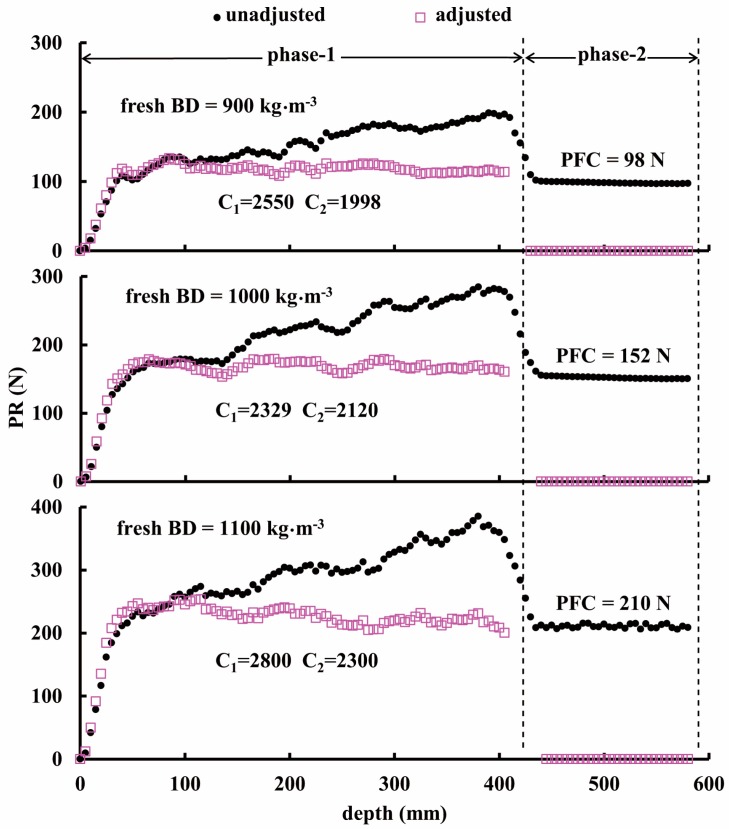
Results of penetrating chopped maize with different packed densities: (**a**) 900 kg·m^−3^, (**b**) 1000 kg·m^−3^, and (**c**) 1100 kg·m^−3^. Solid dots denote uncorrected PR data, hollow squares denote corrected PR data, i.e., PR_c_.

The three graphs in Figure 8 show the PR profiles measured in the maize silage in the cylinders at different levels of fresh BD, i.e., 900, 1000 and 1100 kg·m^−3^. Each graph has two traces associated with the penetration depth; solid dots referring to the PR measurements and the hollow squares to the PR_c_ corrected by the filter (given in Equation (2)). From these graphs, three observations can be clearly made. (i) All of the PR values exhibited a nearly linear relationship with the penetration depth within phase-1. This is because the contact area between the penetration shaft wall and the measured medium increased following the increase of penetration depth [18]; (ii) Within phase-2 the different PFC values became constants, reflecting the effect of BD. In this case the contact area also was constant so that the higher BD packing resulted in the larger PFC [19]; (iii) The optimal values of C_1_ and C_2_ are shown in relation to each BD.

### 3.2. Equation for Transferring PR_c_ to BD

**Figure 9:**
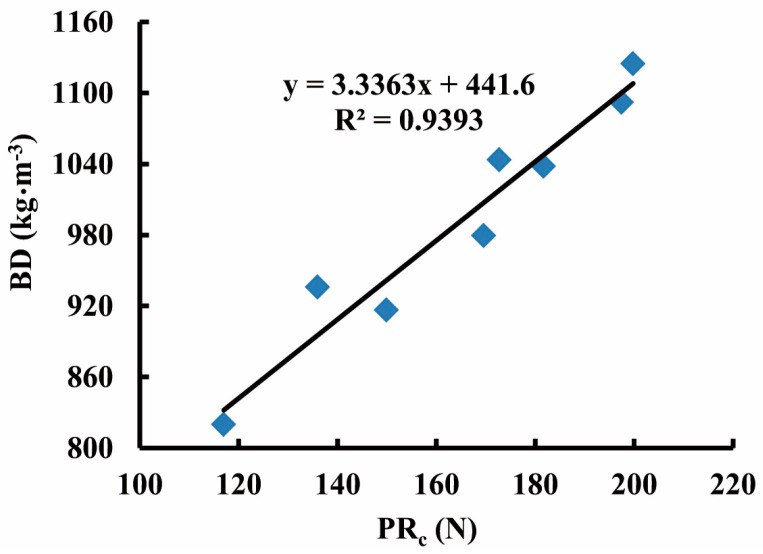
The converting equation between the PR_c_ corrected from penetration resistance (PR) and silage fresh bulk density (BD)

Figure 9 presents a linear regression equation between the values of PR_c_ and the fresh BD values ranging from 820 kg·m^−3^ to 1125 kg·m^−3^ (samples: *n* = 8), which were obtained by the core sampler. The data showing somewhat deviation to the regression line is likely due to the fact that each sample cored in situ had a derivation to the adjacent penetration point as shown Figure 5. Despite this, the high R^2^ (0.9393) suggested the regression equation to be acceptable for converting PR_c_ to BD.

### 3.3. Mapping Silage BD in the Bunker Silo

**Figure 10:**
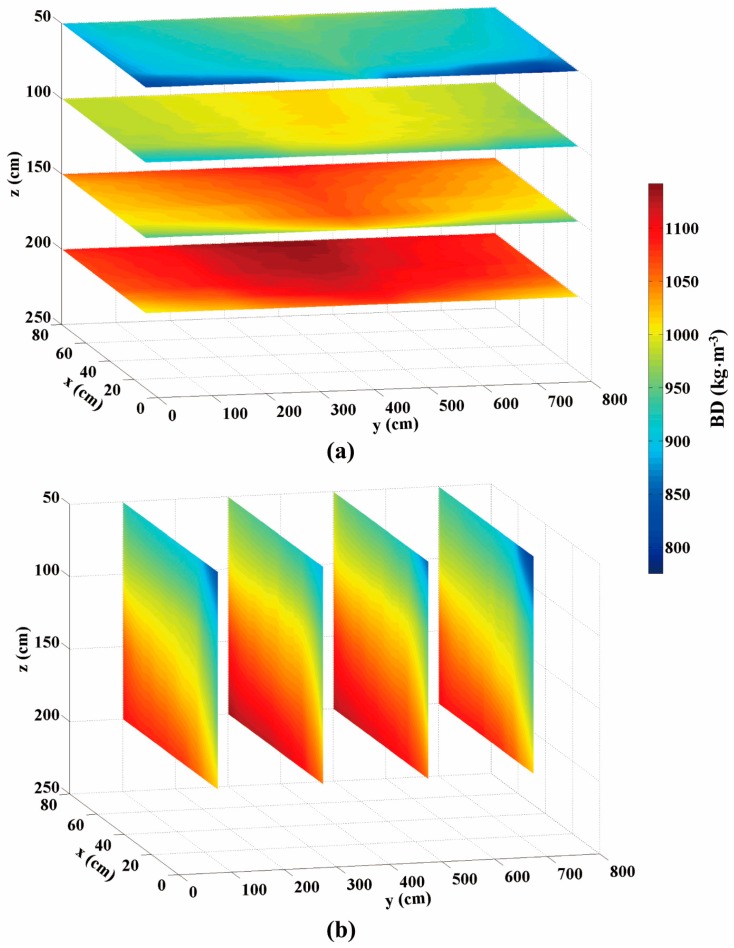
Silage BD maps generated for (**a**) horizontal- and (**b**) vertical-distributions.

**Figure 11:**
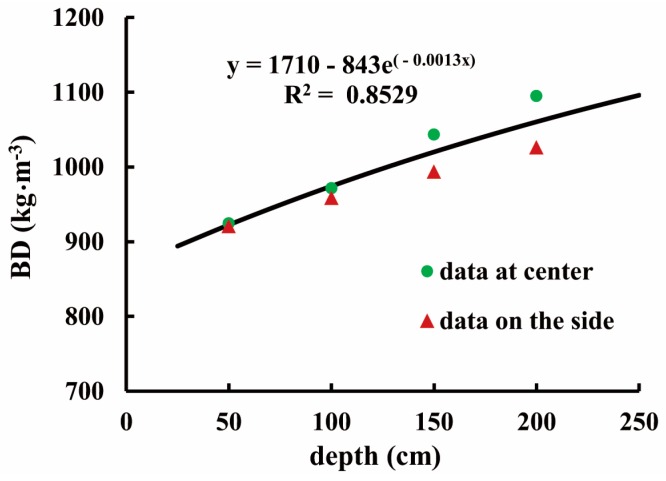
The vertical BD gradient within the bunker silo.

**Figure 12:**
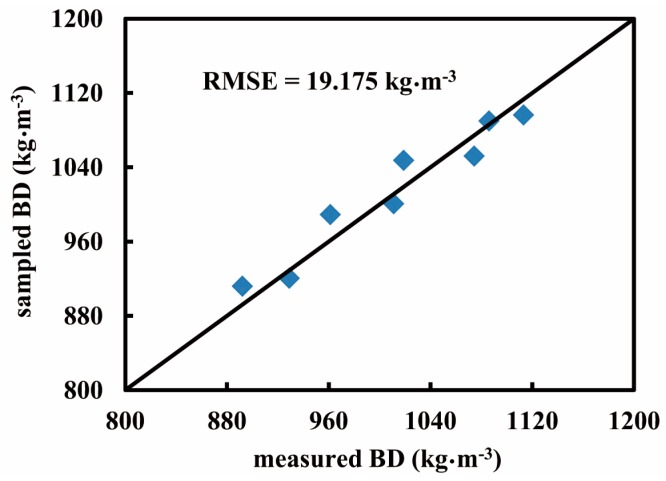
Evaluation of map-based results comparing the core-sampled data with the penetrometer-measured data.

Figure 10 exhibits two groups of BD maps generated from the same volume of the bunker silo. The color bar represents a range of BD varying from 790 to 1120 kg·m^−3^. The upper group (Figure 10a) illustrates slices of the horizontal BD variations and the lower group (Figure 10b) shows vertical BD distributions. More importantly, from each two-dimensional (2D) array we can envision three-dimensional (3D) density distribution. Comparing horizontal with vertical arrays, we see that the horizontal BD exhibited smaller variation, but the vertical BD apparently increased with increasing the vertical depth of the bunker silo (*z*-axis). The average BD near the top layer was 880 kg·m^−3^, whereas that of the bottom was 1090 kg·m^−3^. The increasing gradient of BD along with vertical depth was observed in some previous studies. The literature [8] reported a statistical result surveyed with 175 bunker silos, showing that densities were generally higher in deeper zones. Similarly, another study [20] from 6 maize bunker silos found that cores taken near the top of the silo were always less dense than the samples taken near the floor by an average of 23%. This could be explained due to the effect of self-compaction [8,21,22] or a combination of the self-compaction under silage weight and cumulative compression from the packing tractor [20]. Figure 11 provides the vertical gradient of BD measured from our core data, indicating that self-compaction occurred in this bunker silo as well. In terms of horizontal BD discrepancy, the study [20] reported that samples taken at the center were generally denser than samples taken near the wall by an average of 7%. This is also visible from all maps of Figure 10b. Figure 11 shows similar trends, where the circles denote the core data sampled in the center and the triangles denote core data sampled on the side. Figure 12 shows a comparison with 1:1 line between the map-based BD values (*n* = 8) and the corresponding core-sampled data. The low RMSE (19.175 kg·m^−3^) points to the accuracy of these BD maps, which were generated by the data processing procedures suggested in Figure 4.

## 4. Conclusions

The penetrometer-based bunker silo mapping system coupled with the presented PR data processing procedures, yielded digitally imaged silage BD distributions within the outer 1 m of the exposure face. These horizontal and vertical maps are informative and understandable in relation to the bunker silo and packing characteristics. The agreement between the core sampling data and the map-based results also confirmed the effectiveness of the PFC filter in minimizing the friction noise to the PR measurement. Therefore, the developed penetrometer-based mapping system can potentially contribute to not only detecting poor compaction management, but also in estimating the risk of aerobic deterioration of feeding materials for farm-scale bunker silos.
